# Geotemporospatial and causal inferential epidemiological overview and survey of USA cannabis, cannabidiol and cannabinoid genotoxicity expressed in cancer incidence 2003–2017: part 2 – categorical bivariate analysis and attributable fractions

**DOI:** 10.1186/s13690-022-00812-7

**Published:** 2022-03-30

**Authors:** Albert Stuart Reece, Gary Kenneth Hulse

**Affiliations:** 1 Division of Psychiatry, University of Western Australia, Crawley, Western Australia 6009 Australia; 2 School of Medical and Health Sciences, Edith Cowan University, Joondalup, Western Australia 6027 Australia; 3 Brisbane, Australia

**Keywords:** cannabis, Cannabinoid, Δ9-tetrahydrocannabinol, Cannabigerol, Cannabidiol, Mechanisms, Congenital anomalies, Oncogenesis, Genotoxicity, Epigenotoxicity, Chromosomal toxicity, Multigenerational genotoxicity, Transgenerational teratogenicity, Dose-response relationship, Supra-linear dose response, Sigmoidal dose-response

## Abstract

**Background:**

As the cannabis-cancer relationship remains an important open question epidemiological investigation is warranted to calculate key metrics including Rate Ratios (RR), Attributable Fractions in the Exposed (AFE) and Population Attributable Risks (PAR) to directly compare the implicated case burden between emerging cannabinoids and the established carcinogen tobacco.

**Methods:**

SEER*Stat software from Centres for Disease Control was used to access age-standardized state census incidence of 28 cancer types (including “All (non-skin) Cancer”) from National Cancer Institute in US states 2001–2017. Drug exposures taken from the National Survey of Drug Use and Health 2003–2017, response rate 74.1%. Federal seizure data provided cannabinoid exposure. US Census Bureau furnished income and ethnicity. Exposure dichotomized as highest v. lowest exposure quintiles. Data processed in R.

**Results:**

Nineteen thousand eight hundred seventy-seven age-standardized cancer rates were returned. Based on these rates and state populations this equated to 51,623,922 cancer cases over an aggregated population 2003–2017 of 124,896,418,350. Fifteen cancers displayed elevated E-Values in the highest compared to the lowest quintiles of cannabidiol exposure, namely (in order): prostate, melanoma, Kaposi sarcoma, ovarian, bladder, colorectal, stomach, Hodgkins, esophagus, Non-Hodgkins lymphoma, All cancer, brain, lung, CLL and breast. Eleven cancers were elevated in the highest THC exposure quintile: melanoma, thyroid, liver, AML, ALL, pancreas, myeloma, CML, breast, oropharynx and stomach. Twelve cancers were elevated in the highest tobacco quintile confirming extant knowledge and study methodology. For cannabidiol RR declined from 1.397 (95%C.I. 1.392, 1.402), AFE declined from 28.40% (28.14, 28.66%), PAR declined from 15.3% (15.1, 15.5%) and minimum E-Values declined from 2.13. For THC RR declined from 2.166 (95%C.I. 2.153, 2.180), AFE declined from 53.8% (53.5, 54.1%); PAR declined from 36.1% (35.9, 36.4%) and minimum E-Values declined from 3.72. For tobacco, THC and cannabidiol based on AFE this implies an excess of 93,860, 91,677 and 48,510 cases; based on PAR data imply an excess of 36,450, 55,780 and 14,819 cases.

**Conclusion:**

Data implicate 23/28 cancers as being linked with THC or cannabidiol exposure with epidemiologically-causal relationships comparable to those for tobacco. AFE-attributable cases for cannabinoids (91,677 and 48,510) compare with PAR-attributable cases for tobacco (36,450). Cannabinoids constitute an important multivalent community carcinogen.

## Background

As communities across the globe are increasingly experiencing a rising influx of cannabis products of many types a pleasant confluence of many events suggests that this is a suitable opportunity to re-investigate the important issue of the extent, impact and implications of cannabis-related carcinogenesis.

It has been known for several years that cannabis is linked with testicular cancer rates and indeed all four studies to have investigated the issue have made positive findings [1–4], with a relative risk of 2.59-fold (95%C.I. 1.60–4.19) [5]. Beyond a simple disease linkage this datum is highly impactful for our understanding of disease mechanisms for two reasons both of which are deserving of close attention. It is well described in the testicular cancer literature that the pathogenesis of testicular cancer begins in utero and is activated by the hormonal surge of puberty so that the preclinical phase of the disease takes place over several decades [6–8]. Patients who smoke cannabis and later contract testicular cancer, whose mean age of incidence is around 34 years, have obviously greatly contracted the preclinical disease course. That is to say that cannabis has aggressively accelerated malignant oncogenic processes from several decades to just a few years. Further the testis houses the male germ cell epithelium so that mutation there necessarily implies heritable mutagenesis potentially transmissible to following generations. This combination of powerful carcinogenesis and transgenerational transmissibility is a most concerning confluence. Similarly several pediatric cancers, including acute myeloid leukaemia (AML), have also been linked with parental cannabis use again demonstrating transgenerational transmissibility of oncogenesis [9–15].

It was recently reported in a geospatial and causal inference study that cannabis is a major driver of the significantly rising US total pediatric cancer rates which have risen 49% 1970–2017 [16]. This is important because what is implied is transgenerational transmission of oncogenesis, exactly as suggested above. Furthermore five major chromosomal anomalies and five major cancers were recently linked with cannabis exposure across USA [17].

Moreover cannabis-related oncogenesis is part of a larger overall story of cannabis-related genotoxicity. Warnings are found on the registered product information and prescribing information for both Epidiolex and Sativex indicating that genotoxicity is an activity of cannabinoids which is widely recognized and accepted by regulators, marketers, distributors and many scientists [18, 19]. It is well established that genotoxicity can be expected to be manifested primarily in increased rates of congenital malformations and cancer incidence [20]. Several cardiac malformations were described by the American Heart Association and American Academy of Pediatrics in a major review in 2007 [21]. However it was recently shown, again in a geospatial and causal inference study, that another common congenital heart defect, atrial septal defect secundum type is also being driven sharply upwards by increased cannabis exposure, which is not occurring uniformly across USA [22]. Description of a new cannabis-related congenital anomaly necessarily implies that our understanding of cannabis teratogenesis is as yet incomplete and indeed we have more to learn in this field. Many congenital anomalies were recently described as being more common in the highest quintile of cannabis using US states [23].

Patterns of cannabinoid consumption are changing rapidly. Cannabis legalization has resulted in not only more children and adults exposed to cannabis [24, 25] but also more people using it more intensely so that the number of people smoking daily or near daily has doubled in USA [26]. And it is well established that the concentration of most cannabinoids has risen dramatically in recent decades [27–29]. Hence more people are smoking stronger cannabis with greater intensity than previously creating a triple convergence of cannabinoid exposure especially in habitual smokers. High concentration “dabs”, highly concentrated oils and waxes and solid cannabinoid “shatter” are widely available in many parts of USA. This very new pattern clearly heralds a new era in cannabis epidemiology so that it is only appropriate that we well understand our recent history and epidemiology in this area. Indeed leading authorities have called for a complete revision of cannabis epidemiology in this new high dose – high intensity – high use paradigm [30]. Of note one widely quoted paper with a null finding on the cannabis cancer link actually omitted high dose cannabis smokers from its analysis by protocol likely amputating the most intriguing and important analytical signal [31].

One of the pillars of the epidemiological link between tobacco and lung cancer is the high odds ratio for smokers who experience a nine-fold elevation in lung cancer risk [32]. The E-Value or expected value is a measure on the relative risk scale of the strength of an association which some unmeasured confounder would require with both the exposure of interest and the outcome of concern to explain away the observed association. It can be calculated from the relative risk ratio or from the output from many common regression models. E-Values have both a point estimate and a 95% lower confidence interval bound [33–35]. The applicable lower E-Value for tobacco-lung cancer is 9.0. Our analysis makes extensive use of E-Values on linear regression equations and rate ratio count data for multiple outcomes [35] as was recently recommended by leading public health authorities [36]. We also considered that it would be useful to explore the formal techniques of causal inference and geotemporospatial regression for selected cancers as appropriate.

Cannabis is not a pure substance but a mixture of many substances. Prior to combustion it has over 400 unique chemicals in it collectively known as cannabinoids [37, 38]. Cannabis contains most of the major carcinogens of tobacco including benzopyrene, anthracyclines and aromatic polycyclic hydrocarbons [31, 37, 38]. THC is a major cannabinoid but cannabidiol is a well described minor constituent. Although cannabidiol currently enjoys a relatively harmless reputation in the popular press due to its relative lack of psychoactivity it has been known for several decades to be damaging to chromosomes, the bases of DNA, mitochondrial metabolism and energy generation and the epigenome [39]. Given that it is so widely available we were especially concerned to ascertain if this supposedly “safe” reputation was borne out by the observed epidemiological trends.

Companion papers examine these relationships as continuous variables [40], in detail in prostate and ovarian cancer [41], and the epidemiology of congenital teratogenesis from a space-time and causal inference perspective [17, 42, 43]. The present paper addresses these issues with variables categorized by quintiles of exposure which allows the calculation of key epidemiological metrics including rate ratios (R.R.), attributable fractions in the exposed (AFE) and population attributable risks (PAR, also known as attributable fractions in the population, AFP). Calculation of such proportions across different substances allows the oncogenicity of the known carcinogens tobacco and alcohol to be directly compared with that of the cannabinoids which is the principle subject of the present enquiry.

## Methods

### Data

The Surveillance, Epidemiology and End Results (SEER) database from the Centres for Disease Control (CDC) Atlanta, Georgia and the National Cancer Institute (NCI) and from the National Program of Cancer Registries (NPCR) and SEER Incidence US Cancer Statistics Public Use Database 2019 submission covering years 2001–2017 using the SEER*Stat software was sourced for rates of age-adjusted cancer rates by state and year and cancer type [44]. This study was focussed on 28 of the most common cancers (listed below). One category, called Al Cancer in this report related to the rate of all non-skin cancers. Drug exposure data for USA by state and year was taken from the National Survey of Drug Use and Health (NSDUH) Restricted-Use Data Analysis System (RDAS) of the Substance Use and Mental Health Data Archive (SAMHDA) held by the Substance Use and Mental Health Services Administration (SAMHSA) 2003–2017 [45]. Thus the overlap period between the cancer and drug exposure datasets was 2003–2017 which therefore became the period of analysis. The parameters taken from this dataset were last month cigarettes, last year alcohol use disorder (AUD), last month cannabis, last year non-medical use of opioid analgesics (Analgesics) and last year cocaine. Quintiles of substance exposure were calculated annually and were numbered from one, the lowest quintile, to five the highest exposure quintile. There were no unexposed groups. Median household income, ethnicity and population by state and year data was sourced directly from the US Census bureau via the tidycensus package [46] in R and linear interpolation was used tom complete missing years. The ethnic categories studies were Caucasian-American, African-American, Hispanic-American, Asian-American, American Indian / Alaska Native (AIAN) and Native Hawaiian / Pacific Islander (NHPI). National cannabinoid concentration data across USA was taken from reports published by the US Drug Enforcement Agency (DEA) for the five cannabinoids Δ9-tetrahydrocannabinol (THC), cannabigerol (CBG), cannabichromene (CBC), cannabinol (CBN), and cannabidiol (CBD) [27–29]. National cannabinoid levels were multiplied by state level cannabis use to provide an estimate of state level exposure. Cannabinoid exposure quintiles were calculated on the whole period considered as a whole. Age adjusted case numbers were derived by multiplying the age-adjusted cancer rate in each state and year by the population of that state and dividing it by 100,000.

### Statistical analysis

Data was processed in R-Studio version 1.3.1093 (2009–2020) based upon R version 4.0.3 (2020-10-10). The Shapiro-Wilks test was used to guide log transformation of covariates where appropriate. Data manipulation was performed using the “dplyr” package in the “tidyverse” [47]. Maps and graphs were drawn in R-Base, ggplot2 and “sf” (simple features) [48] and graphs were drawn using ggplot2 from tidyverse [47, 49]. Some colour palettes employed the viridis and plasma palettes taken from the package “Viridis” [50] and several palettes were originally designed for this project. Bivariate maps were drawn using the colorplaner two way colour matrices [51]. Maps and graphs are all original and have not been published elsewhere. Rate ratios, attributable fraction in the exposed and population attributable risks (also known as attributable fraction in the population) were calculated using “epiR” version 2.0.11 developed by Professor Mark Stevenson and colleagues [52]. The Anova test in R-base was used for models comparison.

### Regression models

Bivariate linear trends were computed with linear regression from R-Base.

### Simultaneous multiple model analysis

Simultaneous multiple model analysis was conducted in the tidyverse package “purrr” [47] using tidy and glance from package “broom” [53] using established nest-map-unnest workflows. This methodology allows a whole long dataset providing data on many cancers to be analyzed in a single analysis run at one time.

### Causal inference

E-values were computed using the R-package “EValue” [54] from count data [33–35]. Minimum E-Values above 1.25 are said to suggest causal relationships [33].

*P* < 0.05 was considered significant throughout.

### Data availability

Data, including R-code, ipw weights and spatial weights have been made available through the Mendeley Data repository online and can be freely accessed at 10.17632/dt4jbz7vk4.1

### Ethics

Ethical approval for this study from the University of Western Australia Human Research Ethics Committee was granted on 7th January 2020 with approval number RA/4/20/7724.

## Results

The cancers upon which we chose to focus our attention were chosen because they were relatively common or because they involved tissues which had been implicated in the literature with cannabinoid activities. For this reason cancers of the male and female reproductive tract were well represented amongst the cancers chosen for the present study. The list in alphabetical order includes tumours of: acute lymphoid leukaemia (ALL), acute myeloid leukaemia (AML), bladder, brain, breast, cervix, chronic lymphoid leukaemia (CLL), chronic myeloid leukaemia (CML), colorectum, oesophagus, Hodgkins lymphoma, Kaposi sarcoma, kidney, liver, lung, melanoma, multiple myeloma, Non-Hodgkins lymphoma, oropharynx, ovary, pancreas, penis, prostate, stomach, testis, thyroid and vulva and vagina combined. Based on 2017 data the 27 cancers chosen comprehended 1,339,737 of the 1,670,227 cancers reported to state cancer registries in that year or 80.21% of all non-melanoma non-skin cancers reported. In addition total non-skin cancer was also included in this list making 28 cancer types in all.

Nineteen thousand eight hundred seventy-seven age-adjusted cancer rates were retrieved from the SEER*Stat State NPCR database. The total age-adjusted number of cancers reviewed across the 28 cancer types was 51,623,922 and the total aggregated population across the period 2003–2017 was 124,896,418,350.

Other papers in this series consider these data analyzed as continuous covariates [40] and detailed analyses [41] respectively.

### Bivariate categorical analysis

**Fig. 1:**
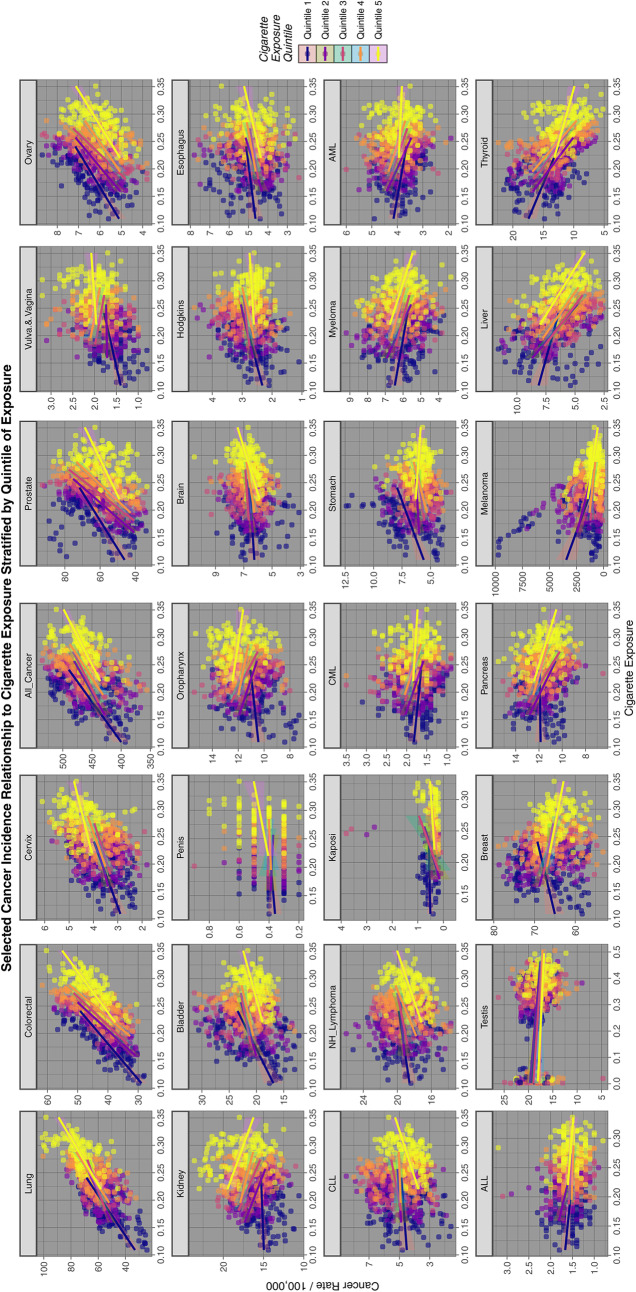
Relationship of selected cancer incidence to tobacco exposure rates by tobacco quintiles

Figure 1 reports graphically a quintile analysis for all cancers for tobacco exposure. The progression by quintile is clearly demonstrated for lung cancer in the first panel and is also evident in different ways for the other tumours displayed.

**Fig. 2:**
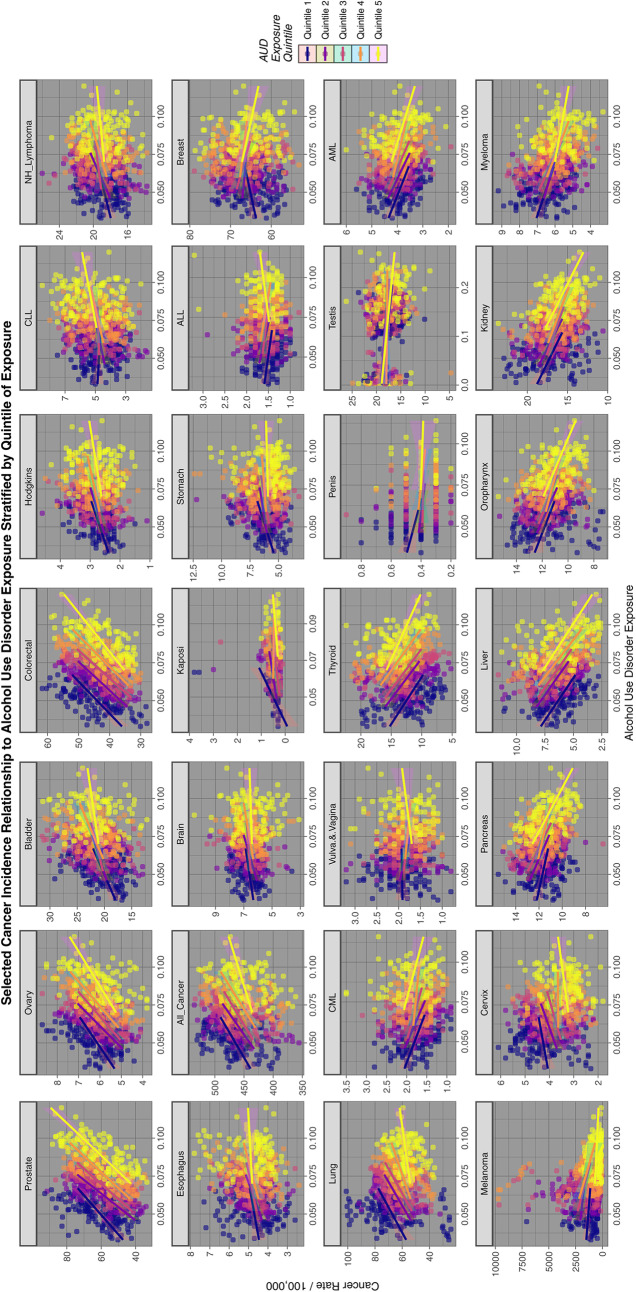
Relationship of selected cancer incidence to AUD exposure rates by AUD quintiles

**Fig. 3:**
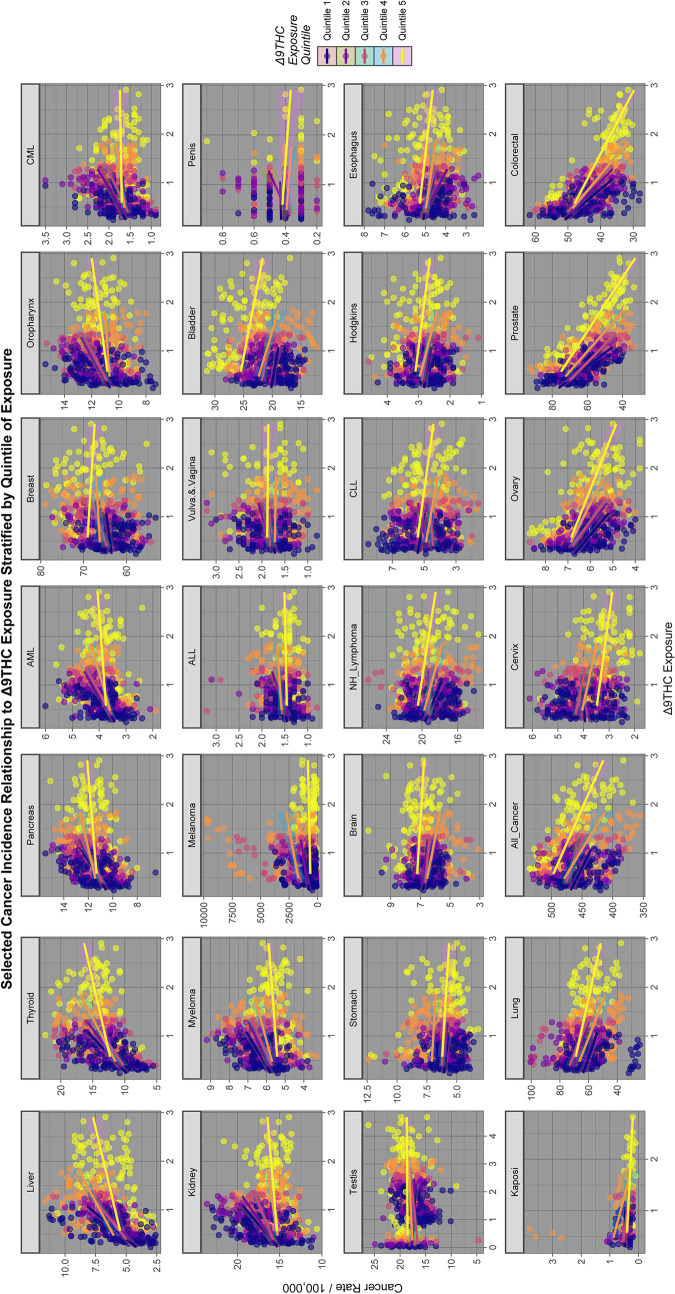
Relationship of selected cancer incidence to THC exposure rates by THC quintiles

**Fig. 4:**
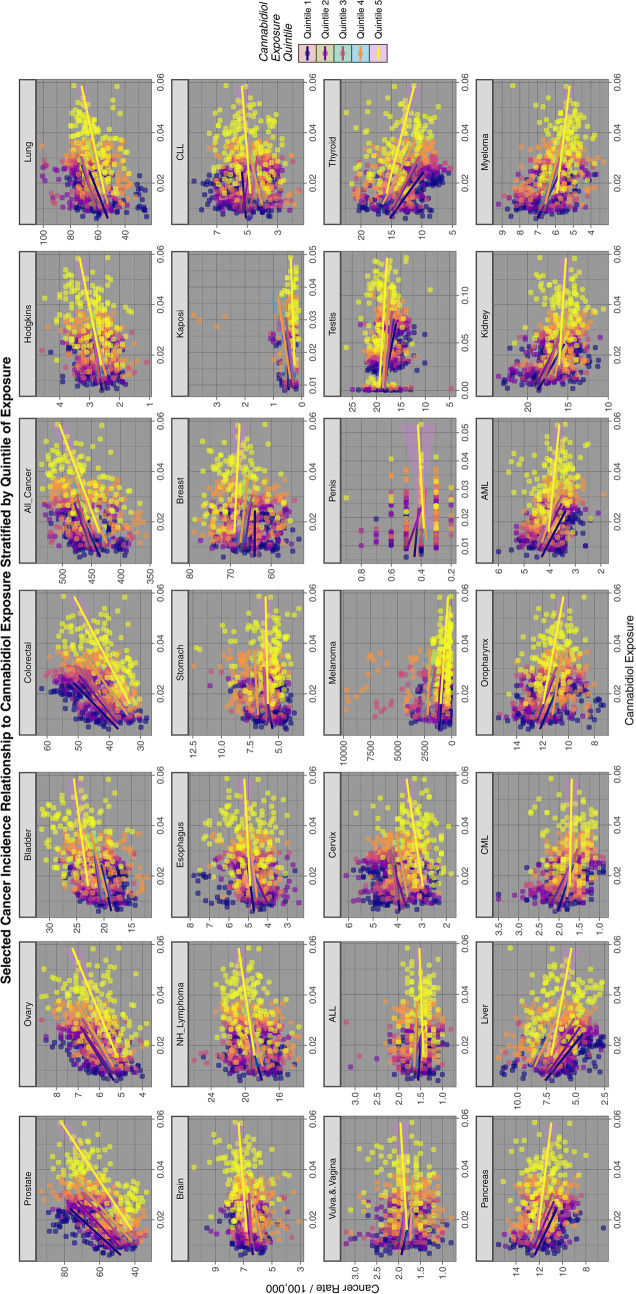
Relationship of selected cancer incidence to cannabidiol exposure rates by cannabidiol quintiles

Figures 2, 3 and 4 perform a similar function for all cancers by AUD, THC and cannabidiol exposure quintiles respectively.

**Fig. 5:**
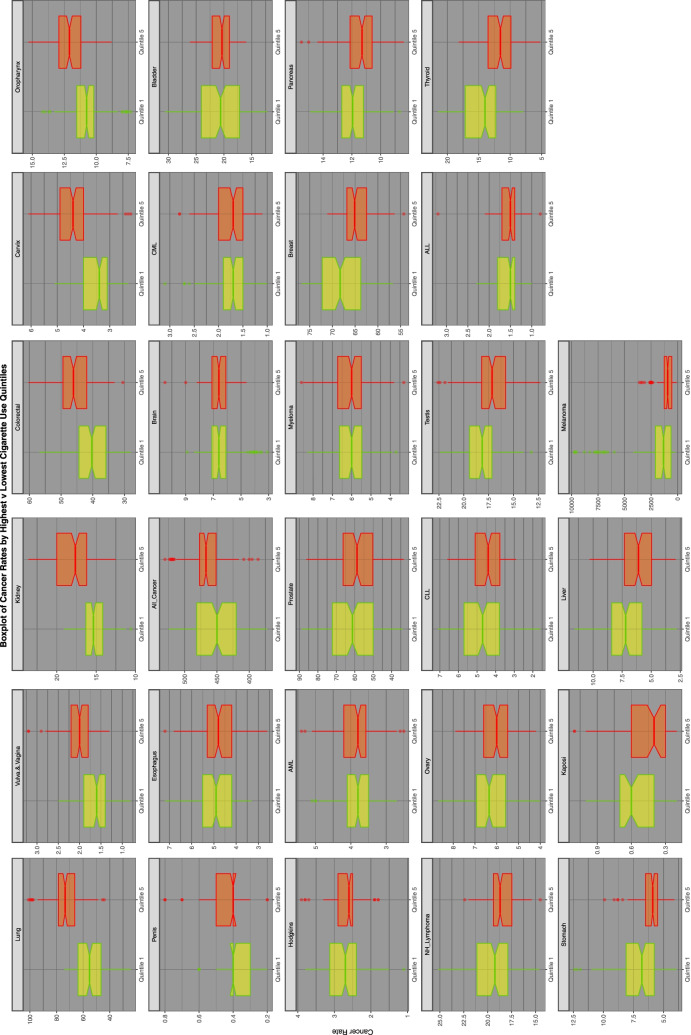
Comparison of lowest and highest quintiles of tobacco exposure on various cancer rates

Figure 5 is a series of boxplots comparing the highest and lowest quintiles’ cancer incidence by tobacco exposure quintile by cancer type. It is ordered by the ratio of the highest to the lowest quintiles. Again lung and vulvovaginal cancers feature at the top of the list.

**Fig. 6:**
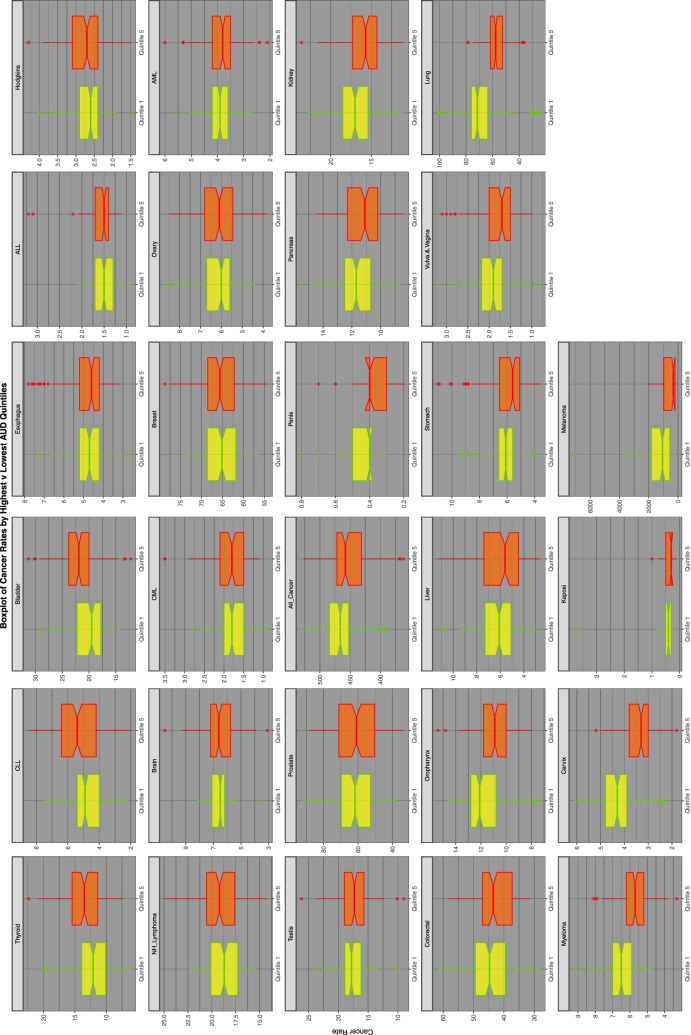
Comparison of lowest and highest quintiles of AUD exposure on various cancer rates

Figure 6 repeats this exercise for AUD exposure quintiles.

**Fig. 7:**
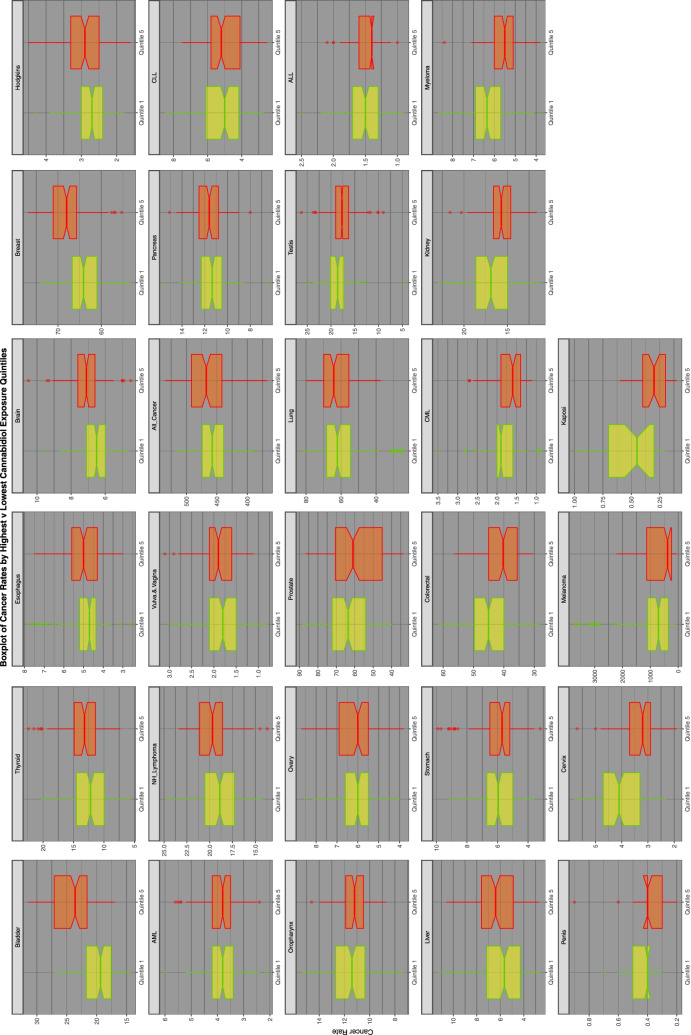
Comparison of lowest and highest quintiles of cannabidiol exposure on various cancer rates

Figure 7 does this for cannabidiol exposure quintiles.

Table 1 presents the quantitative data emerging from these graphs for the comparisons of the highest and lowest tobacco exposure quintiles using the age-adjusted rates and the state population to calculate the expected numbers of cases. This procedure inherently corrects for the differing age structure and therefore cancer predispositions of various state populations. The Table lists the predicted numbers in the highest tobacco using states aggregated over the whole 2003–2017 period, those without cancer, performs similar calculations for the lowest quintile states, presents the rate ratios (RR), the attributable fraction in the exposed (AFE), the population attributable risk (PAR), the applicable *P*-Value and the point estimates and minimum E-Values. In R *P* < 2.2 × 10^− 320^ is the lower limit to which calculations go so P < 2.2 × 10^− 320^ has been inserted in some cells to indicate such vanishingly low significance levels. One notes that 12 cancers in this Table have elevated E-Values. In particular lung, cervix, oropharynx, colorectal, female genital, esophagus, penis, all cancer, CML, kidney and bladder cancer are included on this list which are all known to be associated with tobacco smoking [55].

**Table 1:** Numbers, calculated rates, extreme values, significance and e-values for tobacco

Cancer	Numbers				Calculated Rates					Significance		E-Values	
	Highest Quintile Cancer Count	Highest Quintile Not Cancer Count	Lowest Quintile Cancer Count	Lowest Quintile Not Cancer Count	Highest Quintile State Cancer Rate	Lowest Quitile State Cancer Rate	Rate Ratio (C.I.)	Atrributable Fraction in the Exposed (C.I.)	Population Attributable Risk (C.I.)	Chi Squared	***P***-Value	E-Value - Point Estimate	E-Value - Lower Bound
Lung	656,647	677,158,296	961,061	1,357,237,072	96.9710	70.8101	1.3695 (1.3652, 1.3738)	0.2696 (0.2673, 0.2719)	0.1094 (0.1083, 0.1106)	38,848.04	< 2.2E-320	2.07996	2.07042
Vulva.&.Vagina	29,220	669,780,121	46,922	1,355,478,100	4.3626	3.4616	1.2603 (1.242, 1.2788)	0.2065 (0.1948, 0.218)	0.0792 (0.0741, 0.0844)	967.76	8.12E-213	1.83294	1.79018
Kidney	158,246	677,656,697	273,505	1,357,924,628	23.3520	20.1414	1.1594 (1.1522, 1.1666)	0.1375 (0.1321, 0.1428)	0.0504 (0.0482, 0.0525)	2196.42	< 2.2E-320	1.58920	1.57099
Colorectal	389,377	677,425,566	691,163	1,357,506,970	57.4790	50.9141	1.1289 (1.1245, 1.1334)	0.1142 (0.1107, 0.1176)	0.0411 (0.0398, 0.0425)	3665.82	< 2.2E-320	1.51027	1.49851
Cervix	32,835	677,782,108	58,296	1,358,139,837	4.8445	4.2923	1.1286 (1.1135, 1.144)	0.114 (0.1019, 0.1259)	0.0411 (0.0364, 0.0457)	307.90	2.99E-69	1.50963	1.46890
Oropharynx	115,204	677,699,739	207,304	1,357,990,829	16.9993	15.2655	1.1136 (1.1056, 1.1216)	0.102 (0.0955, 0.1084)	0.0364 (0.0339, 0.0389)	857.67	6.63E-189	1.46916	1.44720
Esophagus	41,573	649,871,778	80,904	1,358,117,229	6.3972	5.9571	1.0739 (1.0612, 1.0867)	0.0688 (0.0577, 0.0797)	0.0233 (0.0194, 0.0273)	139.56	1.34E-32	1.35552	1.31619
Penis	4438	553,731,268	8680	1,190,377,950	0.8015	0.7292	1.0992 (1.0601, 1.1397)	0.0902 (0.0567, 0.1225)	0.0305 (0.0186, 0.0423)	26.28	2.95E-07	1.42936	1.31263
All_Cancer	4,097,024	673,717,919	7,884,694	1,350,313,439	608.1215	583.9159	1.0415 (1.0402, 1.0427)	0.0396 (0.0384, 0.0407)	0.0135 (0.0131, 0.0139)	4422.11	< 2.2E-320	1.24833	1.24383
Brain	53,859	677,761,084	103,713	1,358,094,420	7.9466	7.6366	1.0406 (1.0298, 1.0515)	0.039 (0.0289, 0.049)	0.0133 (0.0098, 0.0168)	56.11	6.85E-14	1.24608	1.20501
Bladder	185,938	677,629,005	366,650	1,357,831,483	27.4395	27.0026	1.0162 (1.0105, 1.0219)	0.0159 (0.0104, 0.0214)	0.0054 (0.0035, 0.0072)	31.77	1.74E-08	1.14438	1.11363
CML	16,596	669,959,025	32,750	1,355,621,500	2.4772	2.4159	1.0254 (1.0064, 1.0447)	0.0248 (0.0064, 0.0428)	0.0083 (0.0021, 0.0145)	6.93	0.008498621	1.18675	1.08680
Hodgkins	18,865	677,796,078	37,284	1,358,160,849	2.7833	2.7452	1.0139 (0.9963, 1.0318)	0.0137 (−0.0037, 0.0308)	0.0046 (− 0.0013, 0.0104)	2.38	0.122840457	1.13251	1.00
AML	40,264	677,774,679	80,681	1,358,117,452	5.9406	5.9407	1 (0.9881, 1.012)	0 (− 0.012, 0.0119)	0 (− 0.004, 0.004)	0.00	0.997775002	1.00414	NA
Prostate	479,151	677,335,792	972,452	1,357,225,681	70.7405	71.6500	0.9873 (0.9839, 0.9907)	−0.0128 (− 0.0164, − 0.0094)	− 0.0042 (− 0.0054, − 0.0031)	52.35	4.64E-13	1.12692	NA
Myeloma	54,005	677,760,938	110,422	1,358,087,711	7.9681	8.1307	0.98 (0.97, 0.9901)	−0.0204 (− 0.031, − 0.01)	−0.0067 (− 0.0101, − 0.0033)	14.80	1.20E-04	1.16470	NA
Breast	576,439	677,238,504	1,180,798	1,357,017,335	85.1161	87.0142	0.9782 (0.9751, 0.9813)	−0.0223 (− 0.0255, − 0.0191)	−0.0073 (− 0.0083, − 0.0063)	188.27	4.37E-43	1.17320	NA
Pancreas	112,830	677,702,113	231,985	1,357,966,148	16.6489	17.0833	0.9746 (0.9677, 0.9815)	−0.0261 (− 0.0334, − 0.0188)	−0.0085 (− 0.0109, − 0.0062)	50.36	1.28E-12	1.18970	NA
NH_Lymphoma	165,126	677,649,817	344,785	1,357,853,348	24.3675	25.3919	0.9597 (0.954, 0.9653)	−0.042 (− 0.0482, − 0.0359)	−0.0136 (− 0.0155, − 0.0117)	189.31	2.64E-43	1.25130	NA
Ovary	52,311	677,762,632	111,212	1,358,086,921	7.7181	8.1889	0.9425 (0.9328, 0.9524)	−0.061 (− 0.0721, − 0.05)	−0.0195 (− 0.0229, − 0.0161)	124.74	2.56E-29	1.31537	NA
CLL	44,694	677,770,249	95,596	1,358,102,537	6.5942	7.0389	0.9368 (0.9264, 0.9474)	−0.0674 (− 0.0795, − 0.0555)	−0.0215 (− 0.0251, − 0.0178)	129.75	2.06E-30	1.33573	NA
Testis	18,973	677,795,970	40,700	1,358,157,433	2.7993	2.9967	0.9341 (0.9182, 0.9504)	−0.0705 (− 0.0891, − 0.0522)	−0.0224 (− 0.028, − 0.0168)	60.10	8.99E-15	1.34525	NA
ALL	11,779	601,068,358	27,611	1,255,633,450	1.9596	2.1990	0.8911 (0.8721, 0.9106)	−0.1221 (− 0.1466, − 0.0982)	−0.0365 (− 0.0432, − 0.0299)	109.78	4.94E-26	1.49237	NA
Thyroid	97,552	677,717,392	225,182	1,357,972,951	14.3941	16.5822	0.868 (0.8616, 0.8746)	−0.152 (− 0.1607, − 0.1434)	−0.0459 (− 0.0483, − 0.0436)	1365.04	3.47E-300	1.57041	NA
Stomach	56,389	677,758,554	136,254	1,358,061,879	8.3199	10.0330	0.8293 (0.8212, 0.8374)	−0.2059 (− 0.2178, − 0.1941)	−0.0603 (− 0.0633, − 0.0572)	1401.96	1.05E-306	1.70413	NA
Kaposi	2824	277,888,455	10,419	794,070,351	1.0161	1.3122	0.7744 (0.7429, 0.8073)	−0.2913 (− 0.3461, − 0.2387)	−0.0621 (− 0.0716, − 0.0527)	146.00	6.53E-34	1.90462	NA
Liver	68,272	677,746,671	178,617	1,358,019,516	10.0733	13.1528	0.7659 (0.7591, 0.7727)	−0.3057 (− 0.3172, − 0.2942)	−0.0845 (− 0.0872, − 0.0819)	3534.82	< 2.2E-320	1.93740	NA
Melanoma	83,366	677,731,577	550,065	1,357,648,068	12.3008	40.5160	0.3036 (0.3014, 0.3058)	−2.2928 (− 2.3169, − 2.2689)	−0.3018 (− 0.303, − 0.3005)	115,616.10	< 2.2E-320	6.04057	NA

Table 2 performs a similar function comparing highest and lowest THC exposure quintiles, with THC quintiles calculated over the whole exposure period in aggregate. 11 cancers in this table have elevated E-Values. Melanoma was most highly significant in this series with rate ratio of 2.16 (95%C.I. 2.15, 2.18), attributable fraction in the exposed 53.83% (53.54, 54.11%), population attributable risk 36.13% (35.87, 36.40%), Chi Squ. = 63,311.55, P < < 2.2 × 10^− 320^, and minimum E-Value 3.73.

**Table 2:** Numbers, calculated rates, extreme values, significance and E-Values for THC

No.	Cancer	Numbers				Calculated Rates					Significance		E-Values	
		Highest Quintile Cancer Count	Highest Quintile Not Cancer Count	Lowest Quintile Cancer Count	Lowest Quintile Not Cancer Count	Highest Quintile State Cancer Rate	Lowest Quitile State Cancer Rate	Rate Ratio (C.I.)	Atrributable Fraction in the Exposed (C.I.)	Population Attributable Risk (C.I.)	Chi Squared	***P***-Value	E-Value - Point Estimate	E-Value - Lower Bound
1	Melanoma	306,838	830,574,345	150,253	881,048,930	36.9429	17.0539	2.1662 (2.1529, 2.1797)	0.5383 (0.5354, 0.5411)	0.3613 (0.3587, 0.364)	63,311.55	2.2E-320	3.75	3.73
2	Thyroid	151,334	830,729,849	107,883	881,091,300	18.2170	12.2442	1.4878 (1.4762, 1.4995)	0.3278 (0.3226, 0.3331)	0.1914 (0.1877, 0.1951)	10,071.61	2.2E-320	2.34	2.31
3	Liver	111,574	830,769,609	82,274	881,116,909	13.4302	9.3375	1.4383 (1.4254, 1.4513)	0.3047 (0.2984, 0.3109)	0.1754 (0.1711, 0.1796)	6324.55	2.2E-320	2.23	2.20
4	AML	52,732	830,828,451	48,622	881,150,561	6.3469	5.5180	1.1502 (1.1361, 1.1645)	0.1306 (0.1198, 0.1412)	0.0679 (0.0619, 0.0739)	496.25	3.11E-110	1.57	1.53
5	ALL	16,966	783,231,729	12,435	647,668,506	2.1662	1.9200	1.1282 (1.1024, 1.1546)	0.1137 (0.0929, 0.1339)	0.0656 (0.053, 0.078)	104.57	7.65E-25	1.51	1.44
6	Pancreas	145,673	830,735,510	139,408	881,059,775	17.5354	15.8228	1.1082 (1.1001, 1.1164)	0.0977 (0.091, 0.1043)	0.0499 (0.0463, 0.0535)	752.96	4.57E-166	1.45	1.43
7	Myeloma	69,035	830,812,148	68,850	881,130,333	8.3093	7.8138	1.0634 (1.0522, 1.0747)	0.0596 (0.0496, 0.0695)	0.0299 (0.0247, 0.035)	130.35	1.73E-30	1.32	1.29
8	CML	20,771	817,987,548	20,592	869,335,404	2.5393	2.3687	1.072 (1.0515, 1.0929)	0.0672 (0.049, 0.085)	0.0337 (0.0243, 0.043)	50.02	1.52E-12	1.35	1.28
9	Breast	725,943	830,155,240	737,913	880,461,270	87.4467	83.8098	1.0434 (1.04, 1.0468)	0.0416 (0.0384, 0.0447)	0.0206 (0.019, 0.0222)	659.85	8.06E-146	1.26	1.24
10	Oropharynx	131,976	830,749,207	137,604	881,061,579	15.8864	15.6180	1.0172 (1.0095, 1.0249)	0.0169 (0.0094, 0.0243)	0.0083 (0.0046, 0.0119)	19.56	9.76E-06	1.15	1.11
**11**	Stomach	76,462	830,804,721	79,792	881,119,391	9.2034	9.0558	1.0163 (1.0063, 1.0264)	0.016 (0.0062, 0.0257)	0.0078 (0.003, 0.0127)	10.21	0.0014	1.15	1.09
12	Kidney	175,942	830,705,241	186,020	881,013,163	21.1798	21.1143	1.0031 (0.9966, 1.0097)	0.0031 (−0.0034, 0.0096)	0.0015 (− 0.0017, 0.0047)	0.87	0.3517	1.06	1.00
13	Testis	99,195	3,340,448,691	9779	329,626,401	2.9695	2.9667	1.001 (0.9804, 1.022)	9E-04 (−0.02, 0.0215)	9E-04 (− 0.0182, 0.0196)	0.01	0.9286	1.03	1.00
14	Vulva.&.Vagina	30,270	818,877,375	32,443	869,064,024	3.6965	3.7331	0.9902 (0.9748, 1.0058)	− 0.0099 (− 0.0258, 0.0058)	−0.0048 (− 0.0124, 0.0028)	1.52	0.1516	1.11	–
15	Penis	5118	705,633,086	5593	741,467,258	0.7253	0.7543	0.9615 (0.9258, 0.9987)	−0.04 (− 0.0802, − 0.0013)	− 0.0191 (− 0.0377, −8E-04)	4.11	0.0252	1.24	–
16	NH_Lymphoma	205,403	830,675,780	221,653	880,977,530	24.7272	25.1599	0.9828 (0.9769, 0.9887)	−0.0175 (− 0.0236, − 0.0114)	−0.0084 (− 0.0113, − 0.0055)	32.07	7.65E-09	1.15	–
17	Bladder	224,188	830,656,995	242,360	880,956,823	26.9892	27.5110	0.981 (0.9754, 0.9867)	−0.0193 (− 0.0252, − 0.0135)	−0.0093 (− 0.0121, − 0.0065)	42.69	3.28E-11	1.16	–
18	Hodgkins	22,119	830,859,064	25,028	881,174,155	2.6622	2.8403	0.9373 (0.9205, 0.9544)	−0.0669 (− 0.0864, − 0.0478)	−0.0314 (− 0.0402, − 0.0227)	49.26	1.14E-12	1.33	–
19	Brain	62,587	830,818,596	70,422	881,128,761	7.5332	7.9922	0.9426 (0.9325, 0.9528)	−0.0609 (− 0.0724, − 0.0496)	−0.0287 (− 0.0339, − 0.0235)	115.98	2.42E-27	1.32	–
20	CLL	56,985	830,824,198	64,593	881,134,590	6.8589	7.3307	0.9356 (0.9252, 0.9462)	−0.0688 (− 0.0809, − 0.0568)	−0.0322 (− 0.0377, − 0.0268)	134.03	2.71E-31	1.34	–
21	Kaposi	4637	479,415,678	3147	235,124,390	0.9672	1.3384	0.7226 (0.6907, 0.7561)	−0.3838 (− 0.4479, − 0.3226)	−0.2286 (− 0.2622, − 0.1959)	199.56	1.31E-45	2.11	–
22	Esophagus	49,631	830,831,552	57,097	872,338,233	5.9737	6.5453	0.9127 (0.9018, 0.9237)	−0.0957 (− 0.1089, − 0.0826)	−0.0445 (− 0.0504, − 0.0387)	221.88	1.77E-50	1.42	–
23	Cervix	33,197	830,847,986	43,411	881,155,772	3.9956	4.9266	0.811 (0.7995, 0.8227)	−0.233 (− 0.2508, − 0.2155)	−0.101 (− 0.1078, − 0.0942)	828.36	1.84E-182	1.77	–
24	Ovary	61,447	830,819,736	76,403	881,122,780	7.3959	8.6711	0.8529 (0.8439, 0.8621)	−0.1724 (− 0.1849, − 0.16)	−0.0768 (− 0.082, − 0.0718)	863.42	4.40E-190	1.62	–
25	All_Cancer	4,669,820	826,211,363	5,337,600	875,861,583	565.2089	609.4114	0.9275 (0.9263, 0.9286)	−0.0777 (− 0.0791, − 0.0764)	− 0.0363 (− 0.0369, − 0.0357)	14,046.15	2.2E-320	1.37	–
26	Colorectal	378,062	830,503,121	545,289	880,653,894	45.5220	61.9186	0.7352 (0.7321, 0.7382)	−0.36 (− 0.3656, − 0.3543)	−0.1474 (− 0.1493, − 0.1454)	21,284.10	2.2E-320	2.06	–
27	Lung	577,790	830,303,393	791,383	880,407,800	69.5878	89.8882	0.7742 (0.7715, 0.7768)	−0.2915 (− 0.2958, − 0.2871)	−0.123 (− 0.1246, − 0.1214)	21,985.04	2.2E-320	1.90	–
28	Prostate	483,411	830,397,772	723,959	880,475,224	58.2144	82.2237	0.708 (0.7054, 0.7106)	− 0.4121 (− 0.4172, − 0.407)	−0.165 (− 0.1667, − 0.1633)	34,883.31	2.2E-320	2.17	–

Table 3 performs a similar function for the upper and lower quintiles of cannabidiol exposure with cannabidiol quintiles calculated over the whole exposure period considered together. 15 cancers in this Table have elevated E-Values. Prostate cancer is most strongly represented with a rate ratio of 1.397 (95%C.I. 1.392, 1.402), attributable fraction in the exposed of 28.40% (28.14, 28.66%) and population attributable risk 15.34% (15.17, 15.51%). Its Chi Squ. value was 32,606.52 at one degree of freedom which corresponds to a *P*-Value << 2.2 × 10^− 320^. The minimum applicable E-Value was 2.13.

**Table 3:** Numbers, calculated rates, extreme values, significance and E-Values for cannabidiol

No.	Cancer	Numbers				Calculated Rates					Significance		e-Values	
		Highest Quintile Cancer Count	Highest Quintile Not Cancer Count	Lowest Quintile Cancer Count	Lowest Quintile Not Cancer Count	Highest Quintile State Cancer Rate	Lowest Quitile State Cancer Rate	Rate Ratio (C.I.)	Atrributable Fraction in the Exposed (C.I.)	Population Attributable Risk (C.I.)	Chi Squared	***P***-Value	E-Value - Point Estimate	E-Value - Lower Bound
1	Prostate	628,831	755,360,319	535,377	898,438,080	83.2491	59.5897	1.397 (1.392, 1.4021)	0.284 (0.2814, 0.2866)	0.1534 (0.1517, 0.1551)	32,606.52	2.2E-320	2.14	2.13
2	Melanoma	214,839	755,774,311	186,976	898,786,481	28.4263	20.8032	1.3664 (1.358, 1.3749)	0.2681 (0.2636, 0.2726)	0.1434 (0.1405, 0.1462)	9821.70	2.2E-320	2.07	2.06
3	Kaposi	5120	402,044,093	1906	201,510,515	1.2735	0.9459	1.3464 (1.2774, 1.4191)	0.2573 (0.2172, 0.2953)	0.1875 (0.1557, 0.218)	123.78	4.75E-29	2.03	1.87
4	Ovary	66,781	755,922,369	64,493	898,908,964	8.8344	7.1746	1.2313 (1.2181, 1.2447)	0.1879 (0.179, 0.1966)	0.0956 (0.0906, 0.1005)	1425.88	2.49E-312	1.77	1.73
5	Bladder	226,085	755,763,065	227,293	898,746,164	29.9148	25.2900	1.1829 (1.176, 1.1898)	0.1546 (0.1496, 0.1595)	0.0771 (0.0744, 0.0797)	3203.50	2.2E-320	1.65	1.63
6	Colorectal	433,034	755,556,116	437,615	898,535,842	57.3133	48.7031	1.1768 (1.1719, 1.1817)	0.1502 (0.1466, 0.1537)	0.0747 (0.0727, 0.0766)	5777.63	2.2E-320	1.63	1.62
7	Stomach	75,022	755,914,128	76,657	898,896,800	9.9247	8.5279	1.1638 (1.1521, 1.1756)	0.1407 (0.132, 0.1493)	0.0696 (0.065, 0.0742)	873.91	2.30E-192	1.60	1.57
8	Hodgkins	22,571	755,966,579	24,142	898,949,315	2.9857	2.6856	1.1118 (1.0918, 1.1321)	0.1005 (0.084, 0.1167)	0.0486 (0.0402, 0.0569)	131.04	1.22E-30	1.46	1.41
9	Esophagus	48,540	755,940,610	52,144	885,656,175	6.4211	5.8876	1.0906 (1.0772, 1.1042)	0.0831 (0.0717, 0.0943)	0.0401 (0.0343, 0.0458)	189.27	2.31E-43	1.40	1.37
10	NH_Lymphoma	197,820	755,791,330	217,228	898,756,229	26.1739	24.1698	1.0829 (1.0763, 1.0895)	0.0765 (0.0709, 0.0822)	0.0365 (0.0337, 0.0393)	657.13	3.15E-145	1.38	1.36
11	All_Cancer	4,624,006	751,365,144	5,120,676	893,852,781	615.4140	572.8769	1.0743 (1.0729, 1.0756)	0.0687 (0.0676, 0.0699)	0.0326 (0.032, 0.0332)	12,396.64	2.2E-320	1.36	1.35
12	Brain	61,078	755,928,072	67,967	898,905,490	8.0799	7.5611	1.0686 (1.057, 1.0804)	0.0642 (0.0539, 0.0744)	0.0304 (0.0254, 0.0354)	141.71	5.67E-33	1.34	1.30
13	Lung	613,629	755,375,521	692,710	898,280,747	81.2350	77.1151	1.0534 (1.0498, 1.0571)	0.0507 (0.0474, 0.0539)	0.0238 (0.0222, 0.0254)	880.93	6.87E-194	1.29	1.28
14	CLL	57,004	755,932,146	65,126	898,908,331	7.5409	7.2450	1.0408 (1.0292, 1.0526)	0.0392 (0.0284, 0.05)	0.0183 (0.0131, 0.0234)	48.70	2.98E-12	1.25	1.20
**15**	Breast	663,949	755,325,201	765,406	898,208,051	87.9024	85.2148	1.0315 (1.0282, 1.0349)	0.0305 (0.0274, 0.0337)	0.0142 (0.0127, 0.0157)	342.56	1.52E-12	1.21	1.20
16	ALL	14,432	706,424,364	14,542	698,662,054	2.0430	2.0814	0.9815 (0.9592, 1.0044)	−0.0188 (− 0.0426, 0.0044)	− 0.0094 (− 0.021, 0.0021)	2.52	0.1126	1.16	–
17	Cervix	32,826	755,956,324	40,390	898,933,067	4.3423	4.4931	0.9664 (0.9525, 0.9806)	− 0.0347 (− 0.0499, − 0.0198)	− 0.0156 (− 0.0222, − 0.009)	21.10	4.35E-06	1.22	–
18	Testis	100,691	3,454,899,465	17,319	568,210,402	2.9144	3.0480	0.9562 (0.9409, 0.9717)	− 0.0458 (− 0.0628, − 0.0291)	−0.0391 (− 0.0535, − 0.0249)	29.67	5.13E-08	1.26	–
19	Pancreas	126,380	755,862,770	158,175	898,815,282	16.7200	17.5982	0.9501 (0.9431, 0.9571)	−0.0525 (− 0.0603, − 0.0448)	−0.0233 (− 0.0267, − 0.02)	184.10	8.88E-77	1.29	–
20	Penis	4195	566,547,406	6196	792,274,553	0.7404	0.7821	0.9468 (0.9104, 0.9846)	−0.0562 (− 0.0984, − 0.0156)	−0.0227 (− 0.039, − 0.0066)	7.48	0.0063	1.30	–
21	Vulva.&.Vagina	27,260	740,944,444	34,886	891,986,887	3.6791	3.9110	0.9407 (0.9259, 0.9557)	−0.063 (− 0.08, − 0.0463)	−0.0277 (− 0.0348, − 0.0205)	57.22	3.91E-14	1.32	–
22	AML	42,798	755,946,352	56,422	898,917,035	5.6615	6.2767	0.902 (0.8907, 0.9134)	−0.1086 (− 0.1227, − 0.0948)	−0.0469 (− 0.0526, − 0.0412)	259.15	0.0714	1.46	–
23	Oropharynx	115,052	755,874,098	151,716	898,821,741	15.2211	16.8794	0.9018 (0.8949, 0.9087)	−0.1089 (− 0.1175, − 0.1005)	−0.047 (− 0.0504, − 0.0435)	700.30	2.27E-06	1.46	–
24	Thyroid	109,996	755,879,154	150,180	898,823,277	14.5521	16.7085	0.8709 (0.8642, 0.8777)	−0.1482 (− 0.1571, − 0.1393)	−0.0626 (− 0.0661, − 0.0592)	1214.15	2.64E-08	1.56	–
25	Liver	86,113	755,903,037	118,781	898,854,676	11.3921	13.2147	0.8621 (0.8545, 0.8697)	−0.16 (− 0.1702, − 0.1498)	−0.0672 (− 0.0712, − 0.0633)	1101.47	3.10E-42	1.59	–
26	Myeloma	57,433	755,931,717	80,524	898,892,933	7.5976	8.9581	0.8481 (0.8391, 0.8573)	− 0.1791 (− 0.1917, − 0.1665)	−0.0745 (− 0.0793, − 0.0698)	911.59	0.0035	1.64	–
27	Kidney	149,860	755,839,290	212,711	898,760,746	19.8270	23.6671	0.8377 (0.8322, 0.8433)	−0.1936 (− 0.2016, − 0.1858)	−0.08 (− 0.083, − 0.0771)	2762.42	1.98E-14	1.67	–
28	CML	17,167	738,183,602	25,089	892,477,941	2.3256	2.8112	0.8273 (0.8114, 0.8435)	−0.2088 (− 0.2325, − 0.1856)	−0.0848 (− 0.0934, − 0.0763)	367.62	1.32E-58	1.71	–

**Fig. 8:**
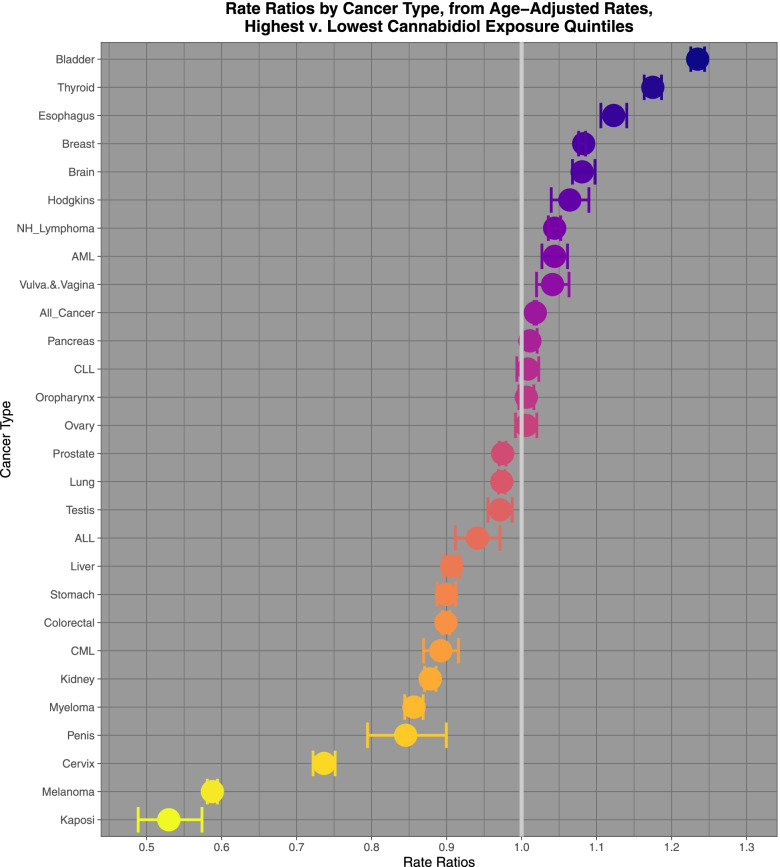
Rate ratios of highest v lowest cannabidiol exposure quintiles calculated from age adjusted rates

Figure 8 sets out the relevant rate ratios (which act like odds ratios for cohort studies) and their tight confidence intervals for cannabidiol exposure.

**Fig. 9:**
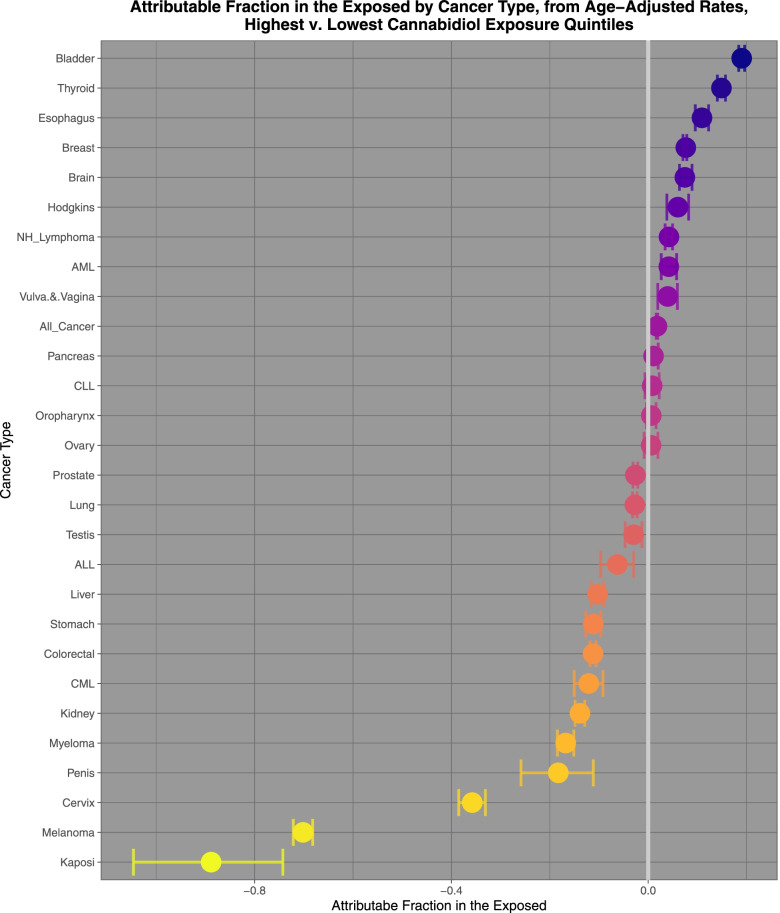
Attributable fractions in the exposed of highest v lowest cannabidiol exposure quintiles calculated from age adjusted rates

Figure 9 sets out the attributable fractions in the exposed and their confidence intervals for cannabidiol exposure. They are noted to decline from almost 20%.

**Fig. 10:**
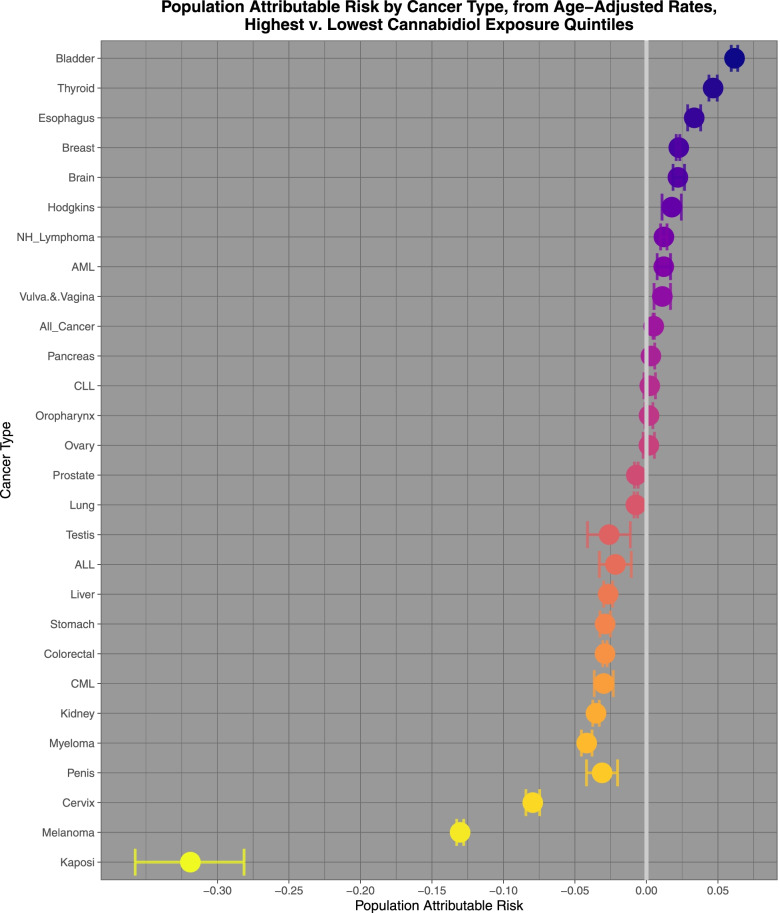
Population attributable risks of highest v lowest cannabidiol exposure quintiles calculated from age adjusted rates

Figure 10 sets out the population attributable risks for the highest and lowest quintiles of cannabidiol exposure.

**Fig. 11:**
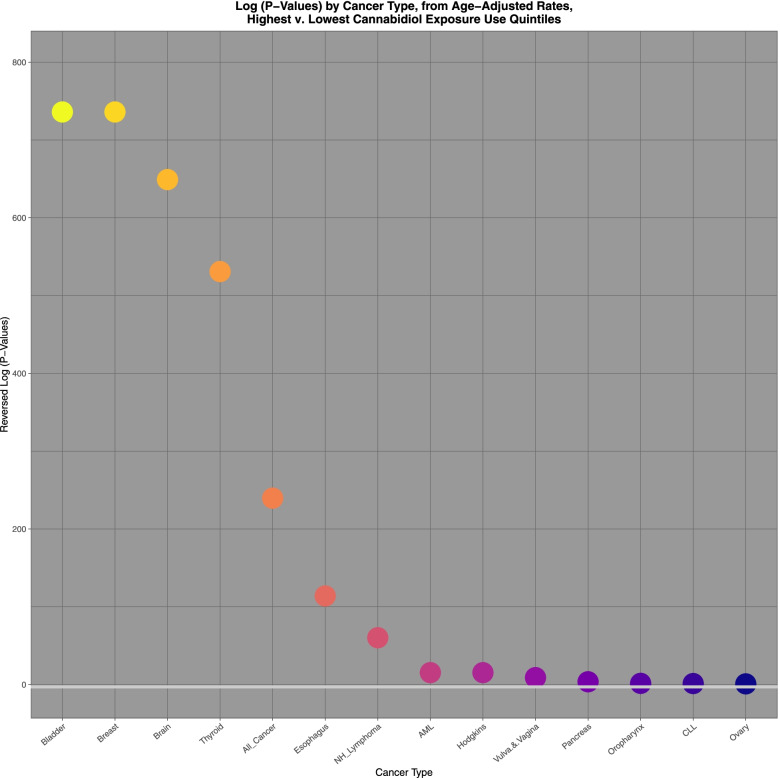
Log P-values ratios of highest v lowest cannabidiol exposure quintiles calculated from age adjusted rates

Figure 11 illustrates graphically the applicable *P*-values for cancers where the risk posed from cannabidiol exposure was elevated and again compares the highest and lowest quintiles. The horizontal line indicates significance on this log scale. The graph may therefore be interpreted as showing illustratively those tumours with elevated P-values for the interquintile comparison.

**Fig. 12:**
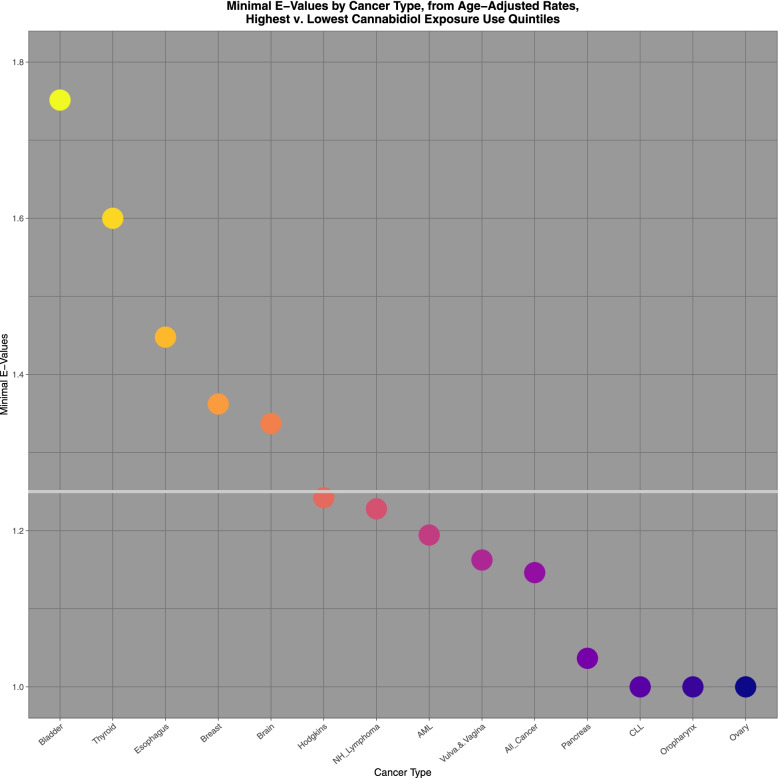
Log E-values ratios of highest v lowest cannabidiol exposure quintiles calculated from age adjusted rates

Figure 12 illustrated the applicable E-Values for these tumours. The horizontal line represents the threshold value of 1.25, which is described in the literature to be indicative of causality [33].

### Summary of bivariate calculations

Finally we turn again to some concluding calculations on the bivariate summary data presented earlier.

Table 4 shows the SEER*Stat derived total case numbers by cancer type for 2017 the final year of the present study. It also shows the attributable fraction in the exposed (AFE) and Population Attributable Risk (PAR) for tobacco, THC and cannabidiol. All the data in the table is complete. The AFE’s and PAR’s are taken from the comparisons listed in Tables 4, 5 and 6.

**Table 4:** Calculated attribtuable fraction in the exposed and population attribtuable risk and case numbers 2017

Cancer	Total Cancers 2017, Surveillance Epidemiology and End Results Data	Cigarettes Attributable Fraction in the Exposed	Cigarettes Population Attributable Risk	THC Attributable Fraction in the Exposed	THC Population Attributable Risk	Cannabidiol Attributable Fraction in the Exposed	Cannabidiol Population Attributable Risk	Cigarette Cancer Numbers - Attributable Fraction in the Exposed	Cigarette Cancer Numbers - Population Attributable Risk	THC Cancer Numbers - Attributable Fraction in the Exposed	THC Cancer Numbers - Population Attributable Risk	Cannabidiol Cancer Numbers - Attributable Fraction in the Exposed	Cannabidiol Cancer Numbers - Population Attributable Risk
Lung	0.2696	0.1094	214,209	−0.2915	−0.1230	− 0.0271	−0.0074	57,749	23,441	−62,434	−26,347	− 5805	− 1585
Vulva.&.Vagina	0.2065	0.0792	6678	−0.0099	−0.0048	0.0397	0.0111	1379	529	−66	−32	265	74
Kidney	0.1375	0.0504	66,500	0.0031	0.0015	−0.1387	−0.0353	9141	3350	206	100	− 9224	− 2347
Colorectal	0.1142	0.0411	139,108	−0.3600	−0.1474	− 0.112	−0.029	15,880	5722	−50,074	−20,503	−15,580	− 4034
Cervix	0.1140	0.0411	12,695	−0.2330	−0.1010	− 0.3577	−0.0795	1447	521	−2958	−1282	− 4541	− 1009
Oropharynx	0.1020	0.0364	45,653	0.0169	0.0083	0.0063	0.0018	4656	1663	771	378	288	82
Penis	0.0902	0.0305	1342	−0.0400	−0.0191	−0.1827	−0.031	121	41	−54	−26	− 245	−42
Esophagus	0.0688	0.0233	16,891	−0.0957	−0.0445	0.1094	0.0334	1162	394	− 1616	− 752	1848	564
All_Cancer	0.0396	0.0135	1,670,227	−0.0777	− 0.0363	0.0179	0.0051	66,096	22,601	−129,830	−60,583	29,897	8518
Brain	0.0390	0.0133	22,127	−0.0609	−0.0287	0.0765	0.0226	863	295	− 1348	−634	1693	500
CML	0.0248	0.0083	6680	0.0672	0.0337	−0.1208	−0.0298	165	56	449	225	−807	−199
Bladder	0.0159	0.0054	74,235	−0.0193	−0.0093	0.1901	0.0616	1182	398	− 1435	−689	14,112	4573
Hodgkins	0.0137	0.0046	8519	−0.0669	−0.0314	0.0604	0.0177	117	39	−570	−267	515	151
AML	0.0000	0.0000	14,928	0.1306	0.0679	0.0423	0.0122	0	0	1949	1014	631	182
Prostate	−0.0128	−0.0042	205,094	−0.4121	− 0.1650	−0.026	− 0.0071	− 2635	−870	−84,517	−33,839	−5332	− 1456
Myeloma	−0.0204	− 0.0067	25,732	0.0596	0.0299	−0.1676	−0.0418	−525	−172	1534	768	− 4313	− 1076
Breast	−0.0223	−0.0073	250,934	0.0416	0.0206	0.0746	0.022	− 5591	−1834	10,427	5171	18,720	5521
Pancreas	−0.0261	−0.0085	48,743	0.0977	0.0499	0.0109	0.0031	− 1272	−416	4760	2432	531	151
NH_Lymphoma	−0.0420	−0.0136	69,718	−0.0175	− 0.0084	0.042	0.0121	− 2930	−949	− 1220	−587	2928	844
Ovary	−0.0610	−0.0195	19,918	−0.1724	− 0.0768	0.0059	0.0017	−1215	−389	− 3434	− 1531	118	34
CLL	−0.0674	−0.0215	16,896	−0.0688	− 0.0322	0.0081	0.0023	− 1139	−363	− 1162	−545	137	39
Testis	−0.0705	−0.0224	10,000	0.0009	0.0009	−0.0297	−0.0261	−705	−224	9	9	−297	−261
ALL	−0.1221	−0.0365	5050	0.1137	0.0656	−0.0626	−0.0217	−617	−184	574	331	−316	−110
Thyroid	−0.1520	−0.0459	45,168	0.3278	0.1914	0.1489	0.0466	− 6865	− 2075	14,807	8645	6726	2105
Stomach	−0.2059	− 0.0603	23,810	0.0160	0.0078	−0.1115	−0.0289	− 4902	−1435	382	187	−2655	−688
Kaposi	−0.2913	−0.0621	849	−0.3838	− 0.2286	−0.8884	− 0.3189	− 247	−53	−326	−194	−754	−271
Liver	− 0.3057	− 0.0845	32,357	0.3047	0.1754	−0.1028	− 0.0268	− 9890	−2735	9860	5675	− 3326	−867
Melanoma	−2.2928	−0.3018	85,362	0.5383	0.3613	−0.7015	−0.1303	−195,722	−25,759	45,949	30,845	−59,881	−11,123

**Table 5:** AFE and PAR calculations by cancer type

Cancer	Total Cancers 2017, Surveillance Epidemiology and End Results Data	Cigarettes Attributable Fraction in the Exposed	Cigarettes Population Attributable Risk	THC Attributable Fraction in the Exposed	THC Population Attributable Risk	Cannabidiol Attributable Fraction in the Exposed	Cannabidiol Population Attributable Risk	Cigarette Cancer Numbers - Attributable Fraction in the Exposed	Cigarette Cancer Numbers - Population Attributable Risk	THC Cancer Numbers - Attributable Fraction in the Exposed	THC Cancer Numbers - Population Attributable Risk	Cannabidiol Cancer Numbers - Attributable Fraction in the Exposed	Cannabidiol Cancer Numbers - Population Attributable Risk
Lung	214,209	0.2696	0.1094	−0.2915	−0.1230	− 0.0271	−0.0074	57,749	23,441				
Vulva.&.Vagina	6678	0.2065	0.0792	−0.0099	−0.0048	0.0397	0.0111	1379	529			265	74
Kidney	66,500	0.1375	0.0504	0.0031	0.0015	−0.1387	−0.0353	9141	3350	206	100		
Colorectal	139,108	0.1142	0.0411	−0.3600	−0.1474	− 0.1120	−0.0290	15,880	5722				
Cervix	12,695	0.1140	0.0411	−0.2330	−0.1010	− 0.3577	−0.0795	1447	521				
Oropharynx	45,653	0.1020	0.0364	0.0169	0.0083	0.0063	0.0018	4656	1663	771	378	288	82
Penis	1342	0.0902	0.0305	−0.0400	−0.0191	−0.1827	−0.0310	121	41				
Esophagus	16,891	0.0688	0.0233	−0.0957	−0.0445	0.1094	0.0334	1162	394			1848	564
All_Cancer	1,670,227	0.0396	0.0135	−0.0777	−0.0363	0.0179	0.0051						
Brain	22,127	0.0390	0.0133	−0.0609	−0.0287	0.0765	0.0226	863	295			1693	500
CML	6680	0.0248	0.0083	0.0672	0.0337	−0.1208	−0.0298	165	56	449	225		
Bladder	74,235	0.0159	0.0054	−0.0193	−0.0093	0.1901	0.0616	1182	398			14,112	4573
Hodgkins	8519	0.0137	0.0046	−0.0669	−0.0314	0.0604	0.0177	117	39			515	151
AML	14,928	0.0000	0.0000	0.1306	0.0679	0.0423	0.0122			1949	1014	631	182
Prostate	205,094	−0.0128	−0.0042	−0.4121	−0.1650	− 0.0260	−0.0071						
Myeloma	25,732	−0.0204	−0.0067	0.0596	0.0299	−0.1676	−0.0418			1534	768		
Breast	250,934	−0.0223	−0.0073	0.0416	0.0206	0.0746	0.0220			10,427	5171	18,720	5521
Pancreas	48,743	−0.0261	−0.0085	0.0977	0.0499	0.0109	0.0031			4760	2432	531	151
NH_Lymphoma	69,718	−0.0420	−0.0136	−0.0175	− 0.0084	0.0420	0.0121					2928	844
Ovary	19,918	−0.0610	−0.0195	− 0.1724	−0.0768	0.0059	0.0017					118	34
CLL	16,896	−0.0674	−0.0215	−0.0688	− 0.0322	0.0081	0.0023					137	39
Testis	10,000	−0.0705	−0.0224	0.0009	0.0009	−0.0297	−0.0261			9	9		
ALL	5050	−0.1221	− 0.0365	0.1137	0.0656	−0.0626	− 0.0217			574	331		
Thyroid	45,168	−0.1520	−0.0459	0.3278	0.1914	0.1489	0.0466			14,807	8645	6726	2105
Stomach	23,810	−0.2059	−0.0603	0.0160	0.0078	−0.1115	−0.0289			382	187		
Kaposi	849	−0.2913	− 0.0621	−0.3838	− 0.2286	−0.8884	− 0.3189						
Liver	32,357	−0.3057	−0.0845	0.3047	0.1754	−0.1028	−0.0268			9860	5675		
Melanoma	85,362	−2.2928	−0.3018	0.5383	0.3613	−0.7015	−0.1303			45,949	30,845		
**Totals**								**93,860**	**36,450**	**91,677**	**55,780**	**48,510**	**14,819**

**Table 6:** Summary Statistics

Substance	2017 Total Cancer Case Numbers	Numbers from Attribtuable Fraction in the Exposed	Numbers from Population Attributable Risk	Percent from Attribtuable Fraction in the Exposed	Percent from Population Attributable Risk
Cigarettes	1,670,227	93,860	36,450	5.62	2.18%
THC	1,670,227	91,677	55,780	5.49%	3.34%
Cannabidiol	1,670,227	48,510	14,819	2.90%	0.89%

Table 5 shows this data again but includes only those tumours with positive AFE’s. It also includes in the last row the applicable totals for the three substances under both AFE and PAR conditions. Clearly the PAR fraction is highly dependent on the penetration of the use of each substance into the community, a factor which is changing rapidly across the USA in relation to cannabinoids. In this respect it is obvious that the PAR for cannabinoids, to which access was until recently relatively restricted, it not properly comparable with that for tobacco and alcohol. This is to say that one cannot properly compare the PAR for licit and illicit substances without careful consideration of the impact of their differing legal statuses on their penetration into the community. It should be noted that the methodology adopted is extremely conservative since the attributable fraction of tobacco for lung cancer in reality is known to be 1.00 [32, 33]. However in the circumstances such an approach is equitable across all substances identified. The number of cases for total cancer has not been included in calculating the column totals, which as shown is 36,450 for tobacco PAR numbers and 48,510 for cannabidiol AFE numbers.

In any event for clarity and for equanimity, the numbers derived from both metrics are presented finally in Table 6. Irrespective of the metric used one notes at once that the numbers of tumours which might be attributable to each substance under these conditions are significant. As mentioned these are clearly highly conservative estimates.

## Discussion

### Main results

When the highest and lowest exposure quintiles were compared 12, 11 and 15 cancers were noted to be elevated in the highest quintiles for tobacco, THC and cannabidiol exposure respectively. Based on 2017 numbers of total non-skin cancer cases (1,670,227) these positively associated cancers translate into an extra 93,860, 91,677 and 48,510 for the three substances on an AFE basis representing 5.62, 5.49 and 2.90% of the total cancer case burden. Based on PAR rates these exposures indicate excess case burdens of 36,450, 55,780 and 14,819 or 2.18, 3.34 and 0.89% respectively. Since cannabis access has until recently been relatively restricted it may be reasonable to compare the PAR rates for legal substances with the AFE rates of the restricted substances THC and cannabidiol, making the cannabinoids important community carcinogens alongside tobacco and alcohol at the population health level.

Comparing the highest and lowest quintiles of THC exposure melanoma, thyroid, liver, AML, ALL, pancreas, myeloma, CML, breast, oropharynx and stomach cancer demonstrated elevated minimum E-Values from 3.72 to 1.08. Rate ratios for these tumours declined from 2.166 (95%C.I. 2.153, 2.180) to 1.016 (1.006, 1.026); AFE declined from 53.8% (53.5, 54.1%) to 1.60% (0.6 to 2.57%); and PAR declined from 36.1% (35.9, 36.4%) to 0.78% (0.30, 0.13%).

Comparing highest and lowest quintiles of cannabidiol exposure prostate, melanoma, Kaposi sarcoma, ovarian, bladder, colorectal, stomach, Hodgkins, esophagus, Non-Hodgkins lymphoma, All cancer, brain, lung, CLL and breast cancer demonstrated elevated minimum E-Values from 2.13 to 1.19. Rate ratios for these tumours declined from 1.397 (95%C.I. 1.392, 1.402) to 1.031 (1.028, 1.035); AFE declined from 28.40% (28.14, 28.66%) to 3.05% (2.74 to 3.37%); and PAR declined from 15.3% (15.1, 15.5%) to 1.42% (1.27, 1.57%).

These general relationships were confirmed with categorical analysis when highest and lowest exposure quintiles were compared. AML, breast, CML, liver, oropharynx, pancreas and thyroid cancers were significantly related to THC exposure when studied as both continuous and categorical variables [40]. All cancers, bladder, brain, breast, colorectal, esophagus, Hodgkins, lung, melanoma, ovary, prostate and stomach cancer were significantly related to cannabidiol exposure when studied both as continuous and categorical variables [40].

### Interpretation

These data suggest that 23 cancers are epidemiologically associated with either THC or cannabidiol with minimum E-values in the same range as those for tobacco. These 23 cancers are: prostate, melanoma, Kaposi sarcoma, ovarian, bladder, colorectal, stomach, Hodgkins, esophagus, Non-Hodgkins lymphoma, All cancer, brain, lung, CLL, breast, thyroid, liver, AML, ALL, pancreas, myeloma, CML, oropharynx.

Based on the numbers of cancers implicated (11 and 15) THC and cannabidiol are as important community carcinogens as tobacco. Based on the case numbers involved THC and cannabidiol are confirmed to be important population health carcinogenic agents particularly if one accepts that it is reasonable to compare the PAR rates for the legal substances with the AFE rates for the restricted substances so that the PAR case numbers of tobacco of 36,450 relate to the AFE numbers of THC and cannabidiol of 91,677 and 48,510. Further, since the E-values for the cannabinoids upon categorical analysis are in the same range as those for tobacco the epidemiological strength of evidence for a causal relationship between the two groups of substance is substantially equivalent. As noted earlier int eh continuous analysis study [40] the evidence for causality is actually stronger for cannabidiol and cannabichromene than for tobacco in that paradigm.

### Mechanisms

The subject of cannabinoids and cancer is too large to be reviewed in detail here. This and related subjects have been described in several other publications to which the interested reader is referred [56–72]. Our intention here is merely to make some observations which are of particular interest and illustrate how all these seemingly disparate observations may present a coherent conceptual framework of cannabinoid-related carcinogenesis.

This section takes the overall plan of first considering the very large field of epigenomics an area which is increasingly being implicated in the pathogenesis of many cancers and also in cannabinoid pathophysiology, and then considering some specific cancers which arise from the above epidemiological analyses. It is intended that this section be read in parallel with the mechanistic sections of the first and third papers in this series.

### Overview of epigenetics

Since the genomic code is the same in each cell the fact that each cell is different implies that the way its complement of genes is used must be different. That is to say control of the available genes is central to cell specification and function. Indeed cell lineage determination is mainly determined by its epigenomic state. The epigenome also carries data on historical exposure to past major events recording neural, immune and metabolic memories [73–80]. Some of the major ways in which epigenomic information is encoded include DNA methylation, post-translational modifications of the tails of the histones around which DNA is wrapped, macro- and micro- RNA’s, position within the cell nucleus in relation to the nuclear membrane, proximity to transcriptional factories also called topologically active domains and whether the gene is subject to major silencing apparatus such as being heavily coated in the repressive machinery as occurs in heterochromatin and the inactivated X-chromosome which becomes the juxta-membrane Barr body. These and other layers of epigenomic machinery do not operate in isolation but are closely coordinated [79, 81, 82].

Epigenomic states including 3-D nuclear spatial organization are heritable across three to four generations [81, 83]. Many organs have been shown to be affected including brain, immunity, obesity, kidney prostate, ovary and testis [65, 66, 71, 81, 84–92]. A variety of phenomena have been shown to be epigenetically inherited including stress, obesity, starvation, the fungicide vinclozin, trauma, chemicals, tobacco, alcohol, opioids, cocaine, and cannabis [64–66, 71, 81, 84, 85, 93, 94].

### DNA methylation

DNA Methylation is a primary mode of control of gene availability. The commonest pattern of aging is that genes become progressively methylated in their promoter regions and demethylated in the gene bodies. This has the overall effect of shutting down gene expression or changing the splice sites or isoforms of transcribed genes. This progressive decline in gene expression clearly fits well with the obvious steady decline in functions as organisms age. It has long been understood that the pattern of DNA methylation at the CpG islands of certain key marker genes can be used to determine an epigenetic age [95–97].

In a recent *tour de force* study from Harvard Aging lab, UCLA and other centres it was shown that reversal of this age-related promoter DNA hypermethylation could actually return the post-mitotic neural cells of the mouse retina to their newborn state and reverse their epigenetic age [98]. This was done by the intraocular delivery of Oct4, Sox2 and Klf4 (OSK) three of the four Yamanaka stem cell inductive factors. Myc was not used as it was not required and has been linked with cancer development. This epigenetic age reversion allowed the ganglion neuronal cells of the retina to recover after a crush injury and to regenerate their axons which were able to grow into the optic chiasm. The acceleration in epigenomic age induced by optic nerve crush injury was reversed by OSK administration and was dependent on the ten-eleven translocation methyldioxygenases (Tet) 1 and 2 which are known to initiate the DNA demethylation process [98]. Accelerated aging of human neurons induced by the chemotherapeutic drug vincristine was similarly reversed by OSK treatment. Murine retinal ganglion cells were also able to regrow and recover after the intraocular hypertension of glaucoma which does not naturally occur including with restoration of impaired sight. They were also able to reverse the aging of advanced mouse retinae, restore the transcriptome to young again and improve sight [98]. Epigenomic gene analysis showed that the most affected genes were special targets of Polycomb Repressive Complex 2 (PRC2) and its histone methyltransferase product trimethylated lysine of histone 3 (H3K27me3). This wonderful bioinformatic approach demonstrates that not only is DNA methylation a hallmark and biomarker of aging but it is also a key cause of the multi-level changes which are known to accompany the aging process.

Cannabis has also been shown to greatly perturb the cellular DNA methylation profile and patterns of both hyper- and hypo- DNA methylation are described with hypomethylation being predominant [64, 65, 71, 84, 85, 93]. Such findings suggest that cannabis exposure may also directly and causally impact the epigenomic aging machinery as has been demonstrated clinically in longitudinal studies [99].

Since aging is the leading risk factor for most adult cancers this would in turn imply a powerful effect widespread across the genome which predisposes towards malignant transformation.

### Histone reduction and modifications

DNA inside cells does not usually occur as long threads but is coiled twice around two sets of four histone proteins which together form a histone octamer with a frequency of around 147 base pairs to form a unit known as a nucleosome. The four histones involved are H2A, H2B, H3 and H4 and two copies each comprise each octamer. This arrangement allows tight packing of DNA and also control over its availability for transcription. Post-translational modifications on the tails of these histones, particularly H3 and H4, control the spacing of the nucleosomes and thus the accessibility of the genes to the transcription machinery.

It was shown by Mon long ago that cannabinoids including THC and cannabinol reduce the synthesis of histones H1, H2A, H2B, H3 and H4 including their acetylated derivatives which make genes available for transcription [100].

If less histones are available for nucleosome casing of DNA it follows that DNA must be less constrained and necessarily inhabit a more open and accessible DNA configuration where it is more accessible to the transcription machinery. This is know to constitute a pro-oncogenic state as stem cell, cell survival and anti-apoptotic genes usually get the upper hand in such situations creating a survival advantage, apoptosis resistance and conferring enhanced clonal replicative capacity. As described below in the discussion on Non-Hodgkins Lymphoma this has been well demonstrated directly for H1 and several of its isoforms.

### Proteins

As catalogued [101] cannabinoids inhibit the synthesis of many proteins. Two of the most important are histones and tubulin which have been discussed above.

### Bioenergetic Epigenomics

Mitochondria are small subcellular organelles within the cytoplasm of all human cells which are known as the “cells powerhouse” as they generate most of the cells energy by oxidative phosphorylation. They also perform several other functions including having a role in cell replication and cell death by apoptosis, antioxidant defence by glutathione maintenance, they protect DNA, and assist with pH and calcium balance and with electrochemical integrity [102].

Mitochondria also carry a full complement of the cannabinoid signalling system. Hence CB1R’s occur in their outer membrane and the intermembrane space and inner mitochondrial membrane actually carry all the machinery necessary to receive and transduce downstream cannabinoid signals [103–110]. It is important to appreciate that as bioactive lipids cannabinoid molecules can pass through lipid-rich biomembranes readily and transmit signals to intracellular sites [105, 108, 111, 112]. In general the action of cannabinoids on mitochondria is inhibitory [105, 108, 111, 112].

Since many reactions involving DNA are energy dependent their continued healthy supply of energy as ATP to the nucleus has major implications for the maintenance of genomic integrity [102].

Mitochondria are involved in epigenomic pathways both directly through the supply of small chemical moieties for post-translational modifications, such as activated phosphate, acetate, methyl, succinate, fumarate, palmitoylation, myristylation and nitrosylation groups but also via coordinated cross-talk and communication channels with the nucleus [113]. Since the mitochondrial DNA codes for many of the mitochondrial proteins, and some are also encoded in the nuclear DNA clearly expression of the two sets of genomes needs to be coordinated. This is fashioned via at least three molecular shuttles involving malate – aspartate, nicotinamide adenine mononucleotide and glyceraldehyde-3-phosphate [113]. For these reasons close relationships between cellular metabolic state and epigenomic systems are well documented and increasingly appreciated as being of importance [74, 78, 113, 114].

### Interactions with specific pathways

Interactions between cannabinoids and many morphogenic pathways have been described. Most of these have been previously implicated in cancer development and malignant transformation. They are discussed further in a companion manuscript [41].

Cannabinoids have been shown to interact with sonic hedgehog [20], fibroblast growth factor ((FGF) [115, 116], including transactivation of the FGF1R by CB1R [117]; bone morphogenetic proteins [118–120], retinoic acid signalling [121–123], notch signalling [124–128] (which is very involved in colorectal cancer), Wnt signalling [129–134] and the hippo pathway [64].

### Generalizability

Our results are likely to be widely generalizable for several reasons. Results presented are internally very consistent both with each other and with much known evidence external to this study. The confirmation of the results for tobacco with those in many other sources is strongly confirmatory both for the tobacco analyses and for the cannabinoids analyses which employ similar methodology [55, 135–139]. The cancer data used are derived from census samples from all US states. The drug exposure data is taken from a well authenticated and widely studied nationally representative survey which has been operating on an annual basis for several decades. The bivariate analysis is at once conceptually simple yet very powerful particularly when paired with E-Value calculations. One of the major result outputs from the present study was E-Values which are a major pillar of causal inference. It was very noteworthy that the E-Values seen for the cannabinoids were of the same order as those for tobacco. We note that the large US dataset represents an ideal context within which to address the present concerns. In that the present results demonstrate causal relationships we are confident that they could be widely reproduced and note that in nations where cannabis use is more widespread we would expect the findings to be more dramatic if the extant data sources are of sufficient quality and currency to properly document the link.

### Strengths and limitations

#### This study a number of strengths

A large national cancer census dataset was used. Age adjusted rates derived from CDC, SEER and NCI were access and employed. The drug dataset was taken from a large well validated nationally representative dataset. The bivariate statistics were straightforward yet, when harnessing the power of E-values they were powerful and enabled us to assess causality directly. These studies were internally and externally consistent with known data both on tobacco-related cancer and on cannabis-related cancer and aetiopathogenesis. Panelled graphs enabled the simultaneous display of results for direct comparison across many different cancer types.

Individual level participant data was not available to this study in common with most epidemiological studies. State-level cannabinoid exposure was estimated as described as state level data itself was not directly available to the present investigators. Another issue of considerable interest is the possible role of synthetic cannabinoids as genotoxins. In the absence of spatiotemporal data on this issue we are unable to comment on this increasingly important matter. However several lines of evidence suggest that they are likely to be implicated. Several recent studies implicate many cannabinoids in genotoxic activities [16, 17, 22, 23, 39, 93, 140–143]. Long ago the genotoxic action was found to reside in the polycyclic olevitol nucleus of the cannabinoids with little modulation by the various side chains [144]. And several other studies implicate synthetic cannabinoids in genotoxicity [145–151]. Overall therefore we feel that this is a fertile and important area for further laboratory based investigation and epidemiological surveillance.

Furthermore this was also an ecological study. It may therefore be seen as potentially being susceptible to the shortcomings typical of ecological studies including the ecological fallacy and selection and information biases. Within the present paper we have carefully addressed such issues with the use of inverse probability weighting in all mixed effects, robust and panel regression models which transform the analytical paradigm from merely an observational study into a pseudorandomized one from which it is entirely appropriate to draw causal inferences. We have also employed E-values widely in many Tables. Therefore these principle tools of quantitative causal analysis have been widely deployed in the present analyses. The issue of causality is further addressed by the detailed pathophysiological mechanisms which have been described above and by mention of other countries where many of the same findings have been made. We therefore feel that we have taken all reasonable steps to minimize observational and ecological shortcomings for prostatic and ovarian cancers and in doing so have demonstrated in a pathfinding way the manner in which such analyses may be extended to other tumours and indeed to other disorders.

## Conclusion

In conclusion this overview of 28 selected cancers showed strong bivariate evidence that THC and cannabidiol were associated with multiple cancers. All cancer incidence was associated with cannabidiol exposure. Breast cancer, the commonest cancer, was associated with tobacco, THC and cannabidiol exposure. 11 cancers were associated with THC and 15 with cannabidiol and together these two cannabinoids alone accounted for 23/28 cancers. The strength of association as measured by the minimum E-Values was equivalent to that from tobacco. The results for tobacco were closely concordant with multiple reports and CDC data an important finding which not only confirms the analysis in relation to tobacco but also confirms the methodology employed for the cannabinoid analyses also. The finding that THC AFE’s declined from 53.8% (53.5, 54.1%) and cannabidiol AFE’s declined from 28.40% (28.14, 28.66%) is very concerning indeed as more people across the globe are exposed to cannabinoids and as cannabinoids increasingly make their way into the food chain of USA, Canada, Europe and Australia amongst many other nations. This is particularly so given the well documented pseudo-exponential relationship of the cannabis genotoxic dose response curve documented both in the laboratory and epidemiologically [41]. The evidence presented strongly implies that the generally benign view with which cannabis and cannabinoids are considered is not supported by the weight of extent epidemiological evidence relating to genotoxicity and carcinogenicity, which is fact is most concerning indeed. The present data is further supported by results presented in the continuous data analyses and more detailed multivariable adjusted causal models in companion and related papers [16, 17, 22, 23, 40, 41, 62, 93, 142, 143, 152–155]. The clear implication from the present work and its accompanying reports [40, 41] is that community penetration of cannabinoids should be carefully restricted not only as a matter of public health and safety including importantly integrity of the food chain, but also as a non-negotiable investment in the genomic health and onco-protection of multiple coming generations in a manner precisely analogous to that of all other seriously genotoxic agents. Particular concerns relate to the movement of increasing sections of the community into higher dose ranges of cumulative cannabinoid exposure in the context of exponentiation of genotoxic dose-responses which has now been convincingly demonstrated both in the laboratory and in epidemiological studies of human populations.

## Data Availability

All data generated or analysed during this study are included in this published article and its supplementary information files. Data has been made publicly available on the Mendeley Database Repository and can be accessed from this URL 10.17632/dt4jbz7vk4.1.

## Abbreviations

AEA: Anandamide
AIAN: American Indian / Alaska Native
ALL: Acute Lymphoid Leukaemia
AML: Acute Myeloid Leukaemia
AUD: Alcohol Use Disorder
CB1R: Cannabinoid Type 1 Receptor
CBC: Cannabichromene
CBD: Cannabidiol
CBG: Cannabigerol
CBN: Cannabinol
CDC: Centers for Disease Control, Atlanta, Georgia
cGAS-STING: cytoplasmic GMP-AMP Synthase and the Stimulators of Interferon Gamma
CLL: Chronic Lymphoid Leukaemia
CML: Chronic Myeloid Leukaemia
DEA: Drug Enforcement Agency
E-Value: Expected Value
FOXM1: Forkhead Box M1
GC: Germinal Centre
IDO2: Indoleamine 2,3 Dioxygenase
mEV: Minimum E-Value (2.5% Threshold Level)
NCI: National Cancer Institute
NHL: Non-Hodgkins Lymphoma
NHPI: Native Hawaiian / Pacific Islander
NPCR: National Program of Cancer Registries
NSDUH: National Survey of Drug Use and Health
SAMHDA: Substance Abuse and Mental Health Services Administration
SAMHSA: Surveillance Epidemiology and End Results Program
SEER: Surveillance epidemiology and End Results Program
THC: Δ9-tetrahydrocannabinol
